# Oral Fc-Coupled Preproinsulin Achieves Systemic and Thymic Delivery Through the Neonatal Fc Receptor and Partially Delays Autoimmune Diabetes

**DOI:** 10.3389/fimmu.2021.616215

**Published:** 2021-08-10

**Authors:** Noémie Corcos, Slobodan Culina, Claire Deligne, Cassandra Lavaud, Sylvaine You, Roberto Mallone

**Affiliations:** ^1^Université de Paris, Institut Cochin, CNRS, INSERM, Paris, France; ^2^Assistance Publique Hôpitaux de Paris, Hôpitaux Universitaires de Paris Centre-Université de Paris, Cochin Hospital, Service de Diabétologie et Immunologie Clinique, Paris, France

**Keywords:** autoimmunity, immune tolerance, neonatal Fc receptor (FcRn), type 1 diabetes, preproinsulin, T cells, thymus, vaccination

## Abstract

Tolerogenic vaccinations using beta-cell antigens are attractive for type 1 diabetes prevention, but clinical trials have been disappointing. This is probably due to the late timing of intervention, when multiple auto-antibodies are already present. We therefore devised a strategy to introduce the initiating antigen preproinsulin (PPI) during neonatal life, when autoimmunity is still silent and central tolerance mechanisms, which remain therapeutically unexploited, are more active. This strategy employs an oral administration of PPI-Fc, i.e. PPI fused with an IgG Fc to bind the intestinal neonatal Fc receptor (FcRn) that physiologically delivers maternal antibodies to the offspring during breastfeeding. Neonatal oral PPI-Fc vaccination did not prevent diabetes development in PPI T-cell receptor-transgenic G9C8.NOD mice. However, PPI-Fc was efficiently transferred through the intestinal epithelium in an Fc- and FcRn-dependent manner, was taken up by antigen presenting cells, and reached the spleen and thymus. Although not statistically significant, neonatal oral PPI-Fc vaccination delayed diabetes onset in polyclonal *Ins2* ^-/-^.NOD mice that spontaneously develop accelerated diabetes. Thus, this strategy shows promise in terms of systemic and thymic antigen delivery *via* the intestinal FcRn pathway, but the current PPI-Fc formulation/regimen requires further improvements to achieve diabetes prevention.

## Introduction

Type 1 diabetes (T1D) is an autoimmune disease mediated by autoreactive T cells that recognize antigens of the insulin-producing pancreatic β cells, leading to their destruction (1–3). A major problem in T1D management is its late diagnosis, which typically occurs after a variable period of subclinical, silent autoimmunity, once a significant proportion of β cells have been destroyed. Hence, T1D prevention and treatment should target the underlying autoimmune mechanisms rather than its metabolic consequences, as done today with insulin replacement therapies. However, immunotherapies aimed at blunting β-cell autoimmunity require an excellent safety profile, since T1D mostly affects children and young adults and is not life-threatening in the short term.

β-cell antigen-specific therapies are therefore attractive in light of their selectivity and safety (4). Such therapies are administered in the form of vaccines comprising β-cell antigens and formulated to achieve immune tolerance. Clinical trials exploiting these strategies have however been disappointing. Several attempts have focused on tolerogenic vaccination with insulin (5, 6), since this is the initiating antigen in the non-obese diabetic (NOD) mouse (7, 8) and, likely, also in humans (5). A trial employing intranasal insulin administration to halt autoimmune β-cell destruction in new-onset T1D patients with slowly evolving disease did not result in significant β-cell preservation, despite evidence that insulin-specific immune tolerance was successfully induced (9). These results suggest that earlier interventions may be needed, before significant β-cell loss and prior to autoimmune progression and antigen spreading.

The safety issue is even more critical for prevention trials using insulin vaccination (10), because they target at-risk subjects who are not yet diabetic. Also in this case, trials have been unsuccessful to date. Such trials enrolled at-risk subjects based on their positivity for multiple auto-antibodies (aAbs), which witness an ongoing autoimmune reaction that already involves several antigens (11). Recent studies further suggest that β-cell autoimmunity initiates very early, possibly already during fetal life, as the median age at aAb seroconversion was only 9-18 months in large prospective cohorts of genetically at-risk children (12, 13). In this context, the PRE-POINT trial (14) targeted at-risk children carrying a high HLA-associated genetic risk of disease but with no signs of active autoimmunity (i.e. aAb^‒^) using a daily oral insulin vaccine. This study documented safety and T-cell modifications compatible with tolerance induction.

The perinatal period may offer better opportunities for T1D prevention not only in terms of timing, but also because it is characterized by immune responses to administered antigens that are biased towards tolerogenic outcomes. Indeed, antigen introduction during fetal and neonatal life results in antigen-specific immune tolerance persisting during adulthood (15, 16). A key role in this process is played by thymic central tolerance (17), in which developing T cells are challenged with ectopically expressed self-antigens such as preproinsulin (PPI). Their recognition leads to elimination of autoreactive T effector cells (Teffs) and to positive selection of T regulatory cells (Tregs). This process is very active during the perinatal period and leads to the definition of immunological self that later imprints peripheral immune responses (18). The ‘immune self-image’ shaping the adult peripheral T-cell repertoire in the thymus is however incomplete, because the self-antigen repertoire is only partially expressed (17, 19), and “prunes” but does not eliminate self-reactive T cells (20–22).

Thus, incomplete central tolerance mechanisms posited as the first checkpoint in T1D progression may be rather universal, as islet-reactive CD8^+^ T cells are detected at similar frequencies in T1D and healthy donors (22–24). Nonetheless, thymic escape of PPI-reactive T cells seems to play a key role in T1D progression. First, the NOD mouse model of T1D develops accelerated diabetes when PPI expression is abolished in the thymus *via* insulin-2 gene (*Ins2*) knock-out (25, 26). Second, human *INS* polymorphic variants predispose to T1D by decreasing PPI expression in the thymus (27). However, this knowledge has not translated into therapeutic strategies aimed at boosting central tolerance *ab initio*. All the tolerogenic vaccination approaches explored to date using the subcutaneous, intranasal or oral route targeted exclusively peripheral tolerance mechanisms (4). PPI introduction in the body, including the thymus, during the perinatal period could instead also boost the thymic T-cell selection process and intervene on the very first step in autoimmune progression. Previous reports suggest that it is possible to ‘upgrade’ central tolerance by administering antigens either intra-thymically (28, 29) or in the periphery (30). In the latter case, a key role is played by migratory dendritic cells (DCs) that ferry these antigens to the thymus (31–35). The key issue is to translate this concept into a therapeutically viable strategy, i.e. using a non-invasive administration route applicable during the perinatal period.

We therefore investigated a strategy that exploits the neonatal Fc receptor (FcRn) pathway that physiologically delivers maternal IgG to the offspring - through the placenta during fetal life and through the gut during lactation (36). To this end, we modified PPI by fusing it to the Fc portion of an IgG1, in order to drive its interaction with the FcRn and its transfer through these epithelial barriers (37). We previously validated the transplacental transfer, documenting that Fc-fused antigens intravenously administered to pregnant mice reach the fetal thymus in an FcRn-dependent manner and promote the generation of antigen-specific Tregs and tolerance (38). When applied to T1D mouse models, PPI-Fc transplacental transfer protected the offspring from diabetes development later in life (39). However, a less invasive administration route is desirable for clinical translation. In this respect, the high FcRn expression in the gut epithelium may allow us to exploit the oral route directly in newborns. We therefore asked whether PPI-Fc orally administered during the neonatal period can be transferred through the intestinal epithelium and reach the thymus in order to induce tolerance and protect from diabetes development.

## Materials and Methods

### Recombinant Proteins and Peptides

PPI-Fc and PPI constructs (containing the murine preproinsulin-1 sequence) were previously described (39). An OVA-Fc construct covering the OVA_243-350_ sequence was further developed (**Supplementary Figure 1**). It comprises the OVA_257-264_ and OVA_323-339_ epitopes recognized by K^b^-restricted OT-I and I-A^d^-restricted OT-II T-cell receptor (TCR)-transgenic T cells, respectively. The Fc domains used were from human IgG1. Recombinant proteins were produced using a Baculovirus expression system and purified on protein G columns (for PPI-Fc and OVA-Fc) and nickel columns (for PPI). Peptides OVA_257-264_ (SIINFEKL), OVA_323-339_ (ISQAVHAAHAEINEAGR), PPI_B15-23_ (LYLVCGERG) and PPI_B9-23_ (SHLVEALYLVCGERG) were chemically synthesized at 85% purity (Chinapeptides). Herceptin (Trastuzumab anti-HER2/neu monoclonal antibody; Genentech) was used as IgG1 control. Trastuzumab F(ab’)2 was generated using the F(ab’)2 Preparation Kit (Pierce #44988, ThermoFisher).

### Mice and Oral Treatment

PPI_B15-23_ TCR-transgenic G9Cα^-/-^.NOD (G9C8.NOD) (40) and *Ins2* ^-/-^.NOD mice (25) were kindly provided by F.S. Wong (University of Cardiff, UK) and C. Boitard (Cochin Institute, Paris, France), respectively. *Fcgrt* ^-/-^.C57BL/6 (FcRn^-/-^.B6) mice were from the Jackson Laboratory. All mice were housed in specific pathogen-free conditions. The study was approved by the French Ministry of Research (#4873-2015113018321568).

G9C8.NOD and *Ins2* ^-/-^.NOD mice were force-fed at day 1, days 1, 4 and 8 or days 1, 4, 7 and 10 of life with 30 µl PPI-Fc (50 µg) or equimolar amounts of control proteins (Fc-devoid PPI, OVA-Fc, IgG1) or phosphate-buffered saline (PBS), using a flexible plastic feeding tube (Instech Laboratories). For diabetes induction, 4-week-old G9C8.NOD mice were primed with 50 µg PPI_B15-23_ peptide and 100 µg CpG (Eurogentec), followed by a second identical immunization 15 days later. Diabetes development was monitored by glycosuria and confirmed by glycemia when positive.

### Tissue Preparation

Protocols are described in Supplementary Methods.

### SDS-PAGE and Western Blot

Pellets were lysed in Radio Immuno Precipitation Assay buffer [150 mM NaCl, 1.0% IGEPAL^®^ CA-630, 0.5% sodium deoxycholate, 0.1% sodium dodecyl sulfate (SDS), 50 mM Tris, pH 8.0] and supernatants retrieved after centrifugation at 16,000g at 4°C for 15 min. Total protein lysates (5 µg/each; Pierce BCA Protein Assay Kit, ThermoFisher) were resolved on a 12% SDS–polyacrylamide gels (SDS-PAGE), transferred to polyvinylidene fluoride membranes and blocked overnight at 4°C in 5% milk/Tris-buffered saline-Tween (TBS-T; 20mM Tris pH 7.5, 150mM NaCl, 0.05% Tween-20). Membranes were then washed thrice in TBS-T and probed with primary antibodies: goat anti-mouse FcRn (RRID : AB_10972971; 1/1,000) and mouse anti-heat shock protein 70 (RRID : AB_627761; 1/1,000) overnight at 4°C, then washed thrice in TBS-T. Membranes were subsequently incubated for 30 min at room temperature with horseradish peroxidase-conjugated goat anti-mouse IgG (RRID : AB_2728714; 1/3,000) or rabbit anti-goat IgG (RRID : AB_562588; 1/5,000), and washed thrice in TBS-T. Bound IgG was detected with the ECL Prime Western Blotting System (GE Healthcare).

### Immunofluorescence, Confocal Microscopy, and Enzyme-Linked Immunosorbent Assays

PPI-Fc and PPI proteins were conjugated with Alexa Fluor (AF)680 using a SAIVI Rapid Antibody/Protein labeling kit (Invitrogen) and orally administered to 1-day-old G9C8.NOD or FcRn^-/-^.B6 mice. Fluorescence (whole body and isolated digestive tract and thymus) was detected using Photon Imager RT (Biospacelab) at a 690-nm excitation and 700-nm emission wavelengths, with 50–100 ms exposures. After imaging, organs (digestive tract and thymus) were included in O.C.T. Compound Orange (TissueTek, Sakura) and frozen at ‒80°C. Sections (7 µm) were air-dried, fixed for 10 min with 4% paraformaldehyde solution and permeabilized with 0.5% Triton X-100 solution. After blocking with 10% goat serum, sections were stained with the following antibodies: rat anti-EpCAM (RRID : AB_1089027; 1/750), rabbit anti-Cytokeratin 5 (RRID : AB_10979451; 1/750), hamster anti-CD11c (RRID : AB_467115; 1/1,000), and mouse anti-insulin (RRID : AB_260137; 1/1,000). For secondary detection, sections were incubated with Cy5-labeled goat anti-rat (RRID : AB_2338264) or anti-rabbit IgG (RRID : AB_2338013), an AF488-labeled goat anti-hamster IgG (RRID : AB_2338997) or an AF594-labeled goat anti-mouse IgG antibody (RRID : AB_2338883). Images were acquired with a Leica DMI6000 confocal microscope and analyzed with the Image J software.

At sacrifice, blood was collected from the same mice. Standard curves were obtained by sequential dilutions of PPI-Fc protein. PPI-Fc was captured on plates coated with an anti-insulin antibody (RRID : AB_2126540; 1/600) and detected with a peroxidase-labeled goat anti-human Fc antibody (RRID : AB_2687484; 1/4,000).

### Protein Uptake

Five-day-old G9C8.NOD mice were force-fed with 50 µg of AF647-labeled PPI-Fc (or IgG1) or PPI (or IgG1 F(ab’)_2_). Spleen, thymus and *lamina propria* (LP) were harvested 4, 8 or 24 h later.

### Flow Cytometry

Detection of AF647-labeled proteins was analyzed in LP, spleen and thymus preparations using an antigen-presenting cell (APC) panel (**Supplementary Table 1**). Thymic EpCAM^+^ cells were analyzed using a thymic epithelial cell (TEC) panel (**Supplementary Table 2**). AF647 median fluorescence was calculated on the total positive cell population.

Spleen, mesenteric (MLNs) and pancreatic lymph nodes (PLNs) were harvested from 7-day-old or 4-week-old G9C8.NOD mice orally treated on day 1 of life with PPI-Fc, PPI, OVA-Fc or PBS. Single-cell suspensions were surface-stained with anti-mouse antibodies (T-cell panel, **Supplementary Table 3**), washed in PBS, fixed/permeabilized with Foxp3 Fix/Perm Buffer (BioLegend) and stained for Foxp3.

Cells were acquired on a 16-color BD LSRII Fortessa and analyzed with FlowJo (v10.6.0).

### Antigen Recall Assays

Splenocytes were incubated with increasing concentrations of PPI_B15-23_ peptide along with an APC-coupled anti-CD107a antibody to assess the cytotoxic activity. Brefeldin-A (20 µg/ml) and monensin (4 µM) were added after 1 h. Cells were further incubated for 5 h before extracellular staining. Cells were then washed in PBS, fixed/permeabilized with the BD Cytofix/Cytoperm buffer, washed twice in PBS 0.1% saponin and stained for interferon (IFN)-γ and tumor necrosis factor (TNF)-α in PBS/0.1% saponin. This recall antibody panel is listed in **Supplementary Table 4**.

### Statistical Analysis

All statistical tests were two-tailed and performed using GraphPad Prism 7, as detailed in the legends of each figure. P values <0.05 were considered significant.

## Results

### Oral PPI-Fc Does Not Prevent Diabetes in PPI TCR-Transgenic G9C8.NOD Mice

**Figure 1:**
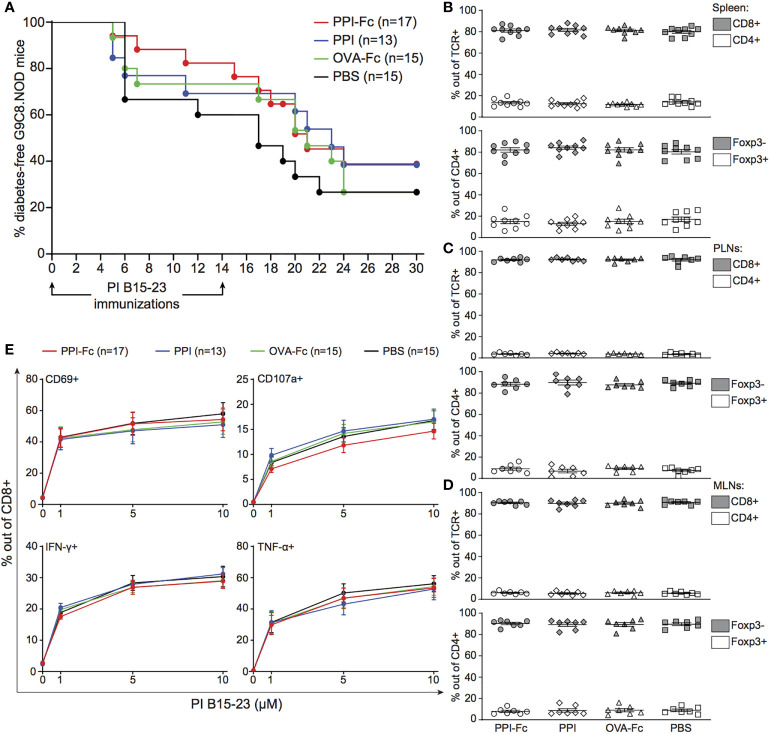
Oral PPI-Fc does not prevent diabetes in TCR-transgenic G9C8.NOD mice. **(A)** Diabetes incidence in G9C8.NOD mice force-fed at day 1 with 50 µg PPI-Fc (red) or equimolar amounts of PPI (blue), OVA-Fc (green) or PBS (black). Diabetes was induced by prime-boost immunization with PI_B15–23_ peptide and CpG at 4 and 6 weeks of age. **(B–E)** Proportion of CD8^+^ and CD4^+^ T cells out of total TCR^+^ cells (top panels) and of Foxp3^‒^ and Foxp3^+^ T cells out of total CD4^+^ T cells (bottom panels) in the spleen **(B)**, PLNs **(C)** and MLNs **(D)** of 4-week-old PPI-Fc- or control-treated G9C8.NOD mice. **(E)** Splenocytes from 4-week-old PPI-Fc- or control-treated G9C8.NOD mice were stimulated *in vitro* with increasing concentrations of PPI_B15-23_ peptide for 5 h and assessed for surface CD69 and CD107a expression and production of IFN-γ and TNF-α. Results are from 4-5 separate experiments with 7-9 mice/group; bars indicate mean ± SEM values.

We previously demonstrated that intravenous vaccination of pregnant G9C8.NOD mice with a single 100-µg dose of PPI-Fc protected the offspring from subsequent diabetes development (39). We therefore tested whether a similar protection was afforded by oral PPI-Fc vaccination directly in newborn mice. To this end, we treated 1-day-old G9C8.NOD mice with a single 50-µg dose of PPI-Fc. This dose was selected by considering the maximal volume compatible with neonatal gavage (30 µl, 1.7 µg/µl), delivery to single rather than multiple pups (as is the case with the 100-µg transplacental delivery), and some degree of gastrointestinal degradation. We then activated the TCR-transgenic T cells of these mice by prime-boost immunization with PPI_B15–23_ peptide and CpG at 4 and 6 weeks of age to induce diabetes (39, 40). As controls, equimolar amounts of recombinant OVA-Fc (i.e., irrelevant protein with preserved FcRn binding), PPI (i.e., cognate antigen with no FcRn binding), or PBS vehicle were orally administered. This oral PPI-Fc treatment did not prevent nor delayed diabetes onset (**Figure 1A**). Similar results were obtained by force-feeding newborn mice with a three-dose 50-µg regimen at day 1, 4 and 8 of life (data not shown).

We verified whether, despite lack of protection, PPI-Fc treatment was able to induce tolerogenic T-cell modifications in the spleen, PLNs and MLNs at 4 weeks of age (i.e. just before PPI_B15-23_ peptide immunization). No difference in CD8^+^, CD4^+^, CD4^+^Foxp3^‒^ T-cell or CD4^+^Foxp3^+^ Treg fractions (**Figures 1B–D**) or absolute numbers (**Supplementary Figure 2**) was observed among treatment groups. Functional analysis of diabetogenic CD8^+^ T cells by *in vitro* peptide recall showed no impairment in the upregulation of activation (CD69) and cytotoxicity (CD107a) markers or in the production of pro-inflammatory cytokines (IFN-*γ*, TNF-α) (**Figure 1E**). Analysis of the LP, spleen and thymus of force-fed G9C8 mice earlier after oral treatment, at day 7 of life, yielded similar findings (**Supplementary Figure 3**).

Collectively, these results show that neonatal oral PPI-Fc vaccination does not prevent diabetes nor it modulates T-cell responses in PPI TCR-transgenic G9C8.NOD mice.

### Oral PPI-Fc Crosses the Gut Epithelium in an FcRn-Dependent Manner and Is Taken up by LP DCs and Macrophages

**Figure 2:**
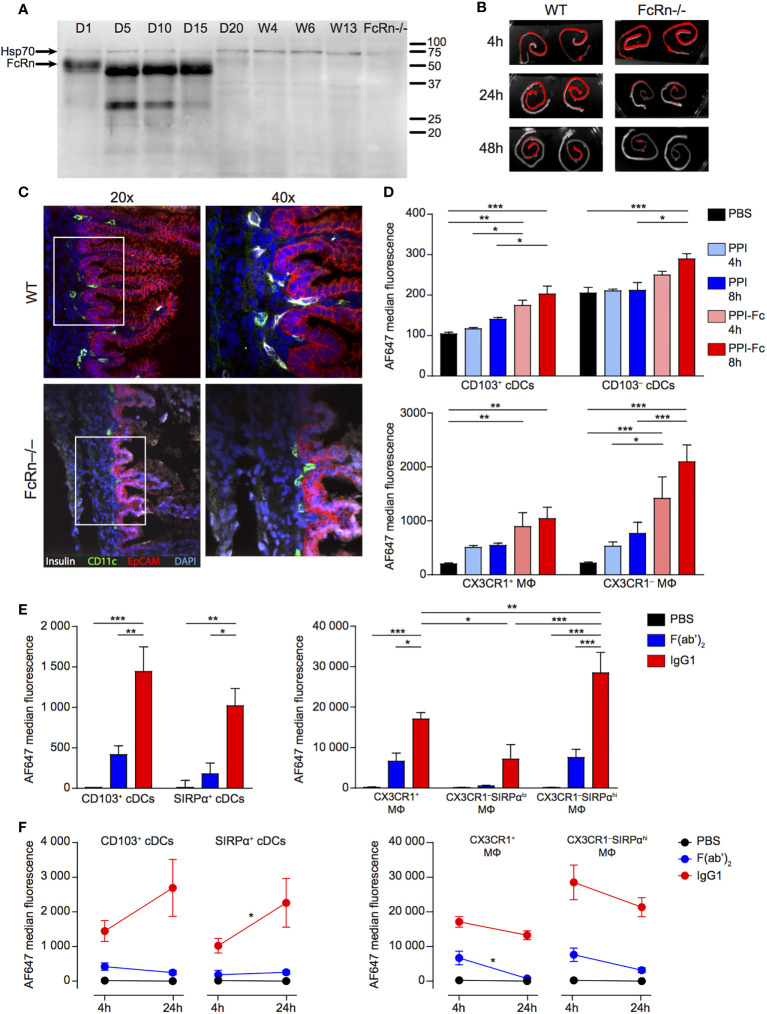
Oral PPI-Fc crosses the gut epithelium in an FcRn-dependent manner and is taken up by LP DCs and macrophages. **(A)** Age-dependent FcRn expression in the gut epithelium. Intestinal epithelial cells were isolated from the whole digestive tract of 1-day-old to 13-week-old G9C8.NOD mice and 1-day-old FcRn^-/-^.B6 mice and probed for FcRn, and Hsp70 as loading control. **(B)** One-day-old wild-type G9C8.NOD (WT) or FcRn^-/-^.B6 mice were force-fed with 50 µg of AF680-labeled PPI-Fc. *Ex-vivo* imaging of the intestines of WT and FcRn^-/-^ mice (n=2/each) 4, 24 or 48 h after PPI-Fc force-feeding. **(C)** Confocal images of the same intestines recovered from WT and FcRn^-/-^.B6 mice at 24 h (20X and 40X magnifications). Sections were stained for insulin (white), CD11c (green) and EpCAM (red), with nuclei counterstained with DAPI (blue). **(D)** Five-day-old G9C8.NOD mice were force-fed with 50 µg AF647-labeled PPI-Fc (red bars), PPI (blue), or PBS (black). LP cells were isolated 4 or 8 h after gavage, and protein uptake (AF647 median fluorescence) was analyzed in CD103^+^ and CD103^‒^ cDCs and CX3CR1^+^CD11b^‒^ and CX3CR1^‒^CD11b^+^ macrophages (MΦ). **(E)** Five-day-old G9C8.NOD mice were force-fed with 50 µg AF647-labeled IgG1 (red), F(ab’)_2_ (blue), or PBS (black). Four hours later, protein uptake was analyzed in the LP for the indicated APC subsets: CD103^+^ and SIRPα^+^ cDCs (left) and CX3XR1^+^, CX3CR1^‒^SIRPα^lo^ and CX3CR1^‒^SIRPα^hi^ macrophages (MΦ, right). **(F)** Comparison of the AF647 median fluorescence at 4 and 24 h in the same APC subsets. Results in **(D–F)** are expressed as mean ± SEM from 3 mice per group. *P ≤ 0.03, **P ≤ 0.007, ***P ≤ 0.0002 by 2-way ANOVA.

Given the absence of any measurable tolerogenic effect, we verified whether orally administered PPI-Fc was able to cross the intestinal epithelium. We first assessed FcRn expression in gut epithelial cells of PPI TCR-transgenic G9C8.NOD mice at different ages by Western blot. FcRn expression was readily detectable at day 1 of life, significantly increased at day 5, 10 and 15, but completely disappeared at day 20 (**Figure 2A**). It remained undetectable in the gut of adult (4-13-week-old) mice, as in 1-day-old FcRn^-/-^.B6 control mice.

We then force-fed G9C8.NOD newborns (wild type, WT) with fluorescent PPI-Fc and analyzed trans-epithelial transfer. *Ex-vivo* gut imaging revealed an intense PPI-Fc signal in both WT and FcRn^-/-^.B6 mice 4 h post-administration, which was maintained over time in WT mice but significantly decreased in FcRn^-/-^.B6 mice already at 24 h, and was barely detectable at 48 h (**Figure 2B**). Confocal microscopy confirmed retention of PPI-Fc signal (counter-stained with an anti-insulin antibody) below the epithelial layer of the small intestine villi (EpCAM^+^, red; **Figure 2C**). Interestingly, PPI-Fc co-localized with the CD11c (green) staining of APCs, and was observed in WT mice but not in FcRn^-/-^.B6 mice.

To better identify the cells responsible for PPI-Fc uptake in newborn mice, we used an antibody panel defining APC sub-populations (**Supplementary Figure 4**). We first compared the uptake of PPI-Fc and Fc-devoid PPI in LP APCs at 4 and 8 h after force feeding (**Figure 2D**). Two subsets of CD11c^hi^ conventional DCs (cDCs) were initially identified, namely CD103^+^ and CD103^‒^. F4/80^+^ macrophages comprised a CX3CR1^+^CD11b^‒^ subset and a CX3CR1^‒^CD11b^+^ subset (CX3CR1^+^ and CX3CR1^‒^ from hereon, respectively). Fluorescent PPI-Fc was detected at both time points in CD103^+^ and CD103^‒^ cDCs and, to a larger extent, in macrophages, especially in the CX3CR1^‒^ subset. The PPI-Fc signal did not increase between 4 and 8 h, and the 4 h time point was retained for further studies. PPI uptake was instead non-significant in all APC populations.

**Figure 3:**
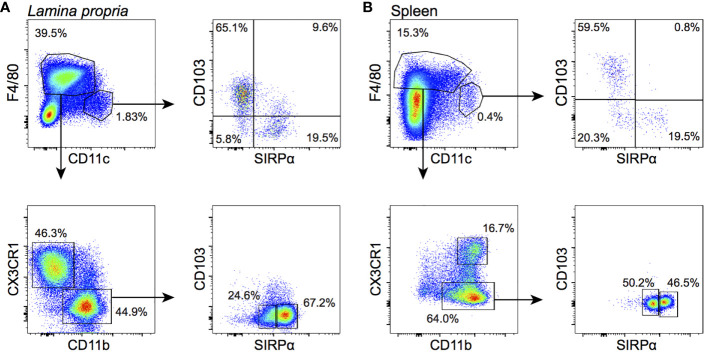
APC subsets in the neonatal LP and spleen. Cells were gated on live CD45^+^Lin^‒^B220^‒^ events (see **Supplementary Figures 3, 4** for details). **(A)** In the LP, CD11c^hi^ cDCs (top left panel) are divided into a CD103^+^ and a SIRPα^+^ subset (top right); F4/80^+^ macrophages are divided into a CX3CR1^+^CD11b^‒^ (indicated as CX3CR1^+^) and a CX3CR1^‒^CD11b^+^ (indicated as CX3CR1^‒^) subset (bottom left). The latter comprises a SIRPα^lo^ and SIRPα^hi^ subpopulation (bottom right). **(B)** In the spleen, CD11c^hi^ cDCs are similarly composed of CD103^+^ and SIRPα^+^ subpopulations. F4/80^+^ macrophages are all CD11b^+^ and either CX3CR1^+^ or CX3CR1^‒^. The latter comprise a SIRPα^lo^ and SIRPα^hi^ subpopulation.

Additional staining for the signal regulatory protein (SIRP)α marker highlighted that the CD103^‒^ cDC subset can be positively defined by SIRPα expression, and we therefore further refer to this CD103^‒^ subset as SIRPα^+^ cDCs (**Figure 3A**). Moreover, CX3CR1^‒^ macrophages could be subdivided into SIRPα^lo^ and SIRPα^hi^. To characterize the 4-h uptake of these refined APC subsets, we used fluorescently-labeled IgG1 and its F(ab’)_2_ form as a proxy for PPI-Fc and PPI, respectively, as these proteins were more readily available and labeled more efficiently. We thus confirmed that the IgG1 uptake by LP CD103^+^ and SIRPα^+^ cDCs was not significantly different (**Figure 2E**, left), as observed for PPI-Fc. More importantly, the SIRPα^hi^ subset accounted for the dominant uptake by CX3CR1^‒^ macrophages (**Figure 2E**, right). Similar to PPI, no significant uptake of F(ab’)_2_ was observed. As the 4-h uptake of IgG1 and F(ab’)_2_ was similarly distributed when compared to that of PPI-Fc and PPI, these proteins were subsequently used to phenotype the APC subsets responsible for the uptake in other organs. We also asked whether this uptake was stable over a longer 24-h time course (**Figure 2F**). While there was a trend toward increased uptake in DCs, the fluorescent signal remained stable and higher in macrophages. In contrast, F(ab’)_2_ uptake remained marginal.

Finally, we investigated the contribution of FcRn to the protein uptake by LP DCs and macrophages of G9C8 newborn mice. In contrast to intestinal epithelial cells, none of these subsets exhibited a significant FcRn expression (**Supplementary Figures 5A, B**), arguing for an FcRn-independent acquisition of PPI-Fc, likely relying on Fc*γ* receptors.

Collectively, these results show that orally administered PPI-Fc crosses the intestinal epithelium in an Fc-FcRn-dependent fashion. It is subsequently taken up by LP CD11c^+^ APCs, both CD103^+^ and SIRPα^+^ cDCs and macrophages (mostly CX3CR1^‒^SIRPα^hi^), and this uptake is not observed for Fc-devoid PPI.

### Oral PPI-Fc Reaches the Spleen Mainly in Soluble Form and Is Taken up Locally by SIRPα^+^ cDCs and Macrophages

**Figure 4:**
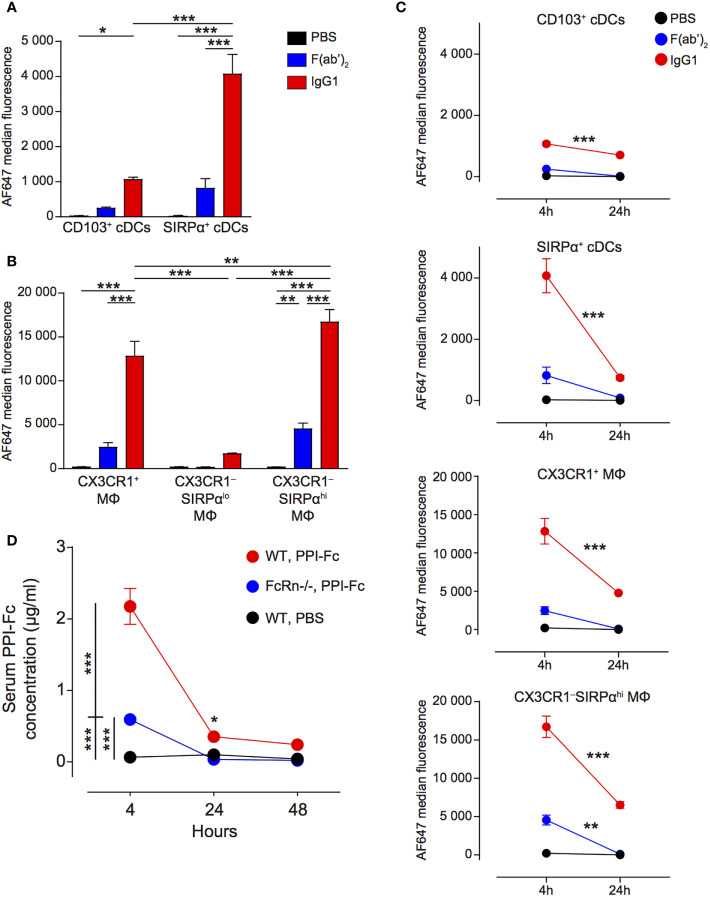
Oral PPI-Fc reaches the spleen mainly in soluble form and is taken up locally by SIRPα^+^ cDCs and macrophages. **(A, B)** Five-day-old G9C8.NOD mice were force-fed with 50 µg AF647-labeled IgG1 (red), F(ab’)_2_ (blue), or PBS (black). Four hours later, protein uptake (AF647 median fluorescence) was analyzed in the spleen for the indicated APC subsets: CD103^+^ and SIRPα^+^ cDCs **(A)** and CX3XR1^+^, CX3XR1^‒^SIRPα^lo^ and CX3XR1^‒^SIRPα^hi^ macrophages **(B)**. **(C)** Comparison of the AF647 median fluorescence at 4 and 24 h in the same APC subsets. **(D)** One-day-old wild-type G9C8.NOD (WT) or FcRn^-/-^.B6 mice were force-fed with 50 µg of PPI-Fc (or PBS in WT mice), serum was collected after 4, 24 and 48 h and PPI-Fc concentrations measured by enzyme-linked immunosorbent assays. Results are expressed as mean ± SEM from 3 mice per group (6/group in **D**). *P ≤ 0.03, **P ≤ 0.006, ***P ≤ 0.0003 by 2-way ANOVA.

Having defined that oral PPI-Fc crosses the gut epithelium and is taken up locally by cDCs and macrophages, we asked whether systemic bioavailability was achieved. To this end, the uptake by splenic APCs was assessed 4 h after gavage. These APCs comprised CD11c^hi^ cDCs, which were less represented than in the LP but could be similarly subdivided into CD103^+^SIRPα^‒^ (CD103^+^) and SIRPα^+^CD103^‒^ (SIRPα^+^) (**Figure 3B**; see **Supplementary Figure 6** for the detailed gating strategy). Although all F4/80^+^ macrophages were here CD11b^+^, CX3CR1^+^ and CX3CR1^‒^ subpopulations were distinguished. The proportion of CX3CR1^‒^ macrophages was higher in the spleen, and could be further subdivided into SIRPα^lo^ and SIRPα^hi^ as in the LP. Contrary to what observed in the LP, IgG1 fluorescence in cDCs was mostly detected in the SIRPα^+^ rather than in the CD103^+^ subset (**Figure 4A**). Also in the spleen, CX3CR1^‒^SIRPα^hi^ and, to a lesser extent, CX3CR1^+^ macrophages accounted for most of the uptake (**Figure 4B**), while F(ab’)_2_ uptake was negligible in all cases. At 24 h, IgG1 fluorescence decreased in CD103^+^ cDCs, and it drastically declined in SIRPα^+^ cDCs and in CX3CR1^+^ and CX3CR1^‒^SIRPα^hi^ macrophages (**Figure 4C**).

Since macrophages are non-migratory APCs and CD103^+^ cDCs (which displayed lower uptake than SIRPα^+^ cDCs and macrophages) are the main subset described as migrating from the gut (41), our observations suggest that most Fc-coupled proteins may travel from the LP to the spleen in soluble form to be taken up locally. We therefore verified whether orally administered PPI-Fc could be detected in the serum of treated mice. Four hours after gavage, serum PPI-Fc was present at a concentration of 2.2 µg/ml in WT G9C8.NOD mice (**Figure 4D**). Serum PPI-Fc concentrations in FcRn^-/-^.B6 mice were instead significantly lower (0.6 µg/ml), but not absent, indicating some passive transfer independent of the FcRn pathway. In line with the kinetics of uptake by non-migratory splenic APCs, PPI-Fc concentration dropped at 24 and 48 h, indicating that the distant uptake of soluble PPI-Fc outside the LP is most efficient within the first hours after oral treatment.

Finally, as in the LP, also in the spleen neither DCs nor macrophages expressed significant levels of FcRn (**Supplementary Figure 5C**), suggesting a preferential protein-Fc uptake through Fc*γ* receptors.

Collectively, these results show that orally administered PPI-Fc reaches the spleen mainly in soluble form and, to a lesser degree, *via* gut-derived CD103^+^ cDCs. It is subsequently taken up locally by SIRPα^+^ cDCs and macrophages.

### Oral PPI-Fc Reaches the Thymus Mainly Through Ferrying by Migratory SIRPα^hi^ cDCs

**Figure 5:**
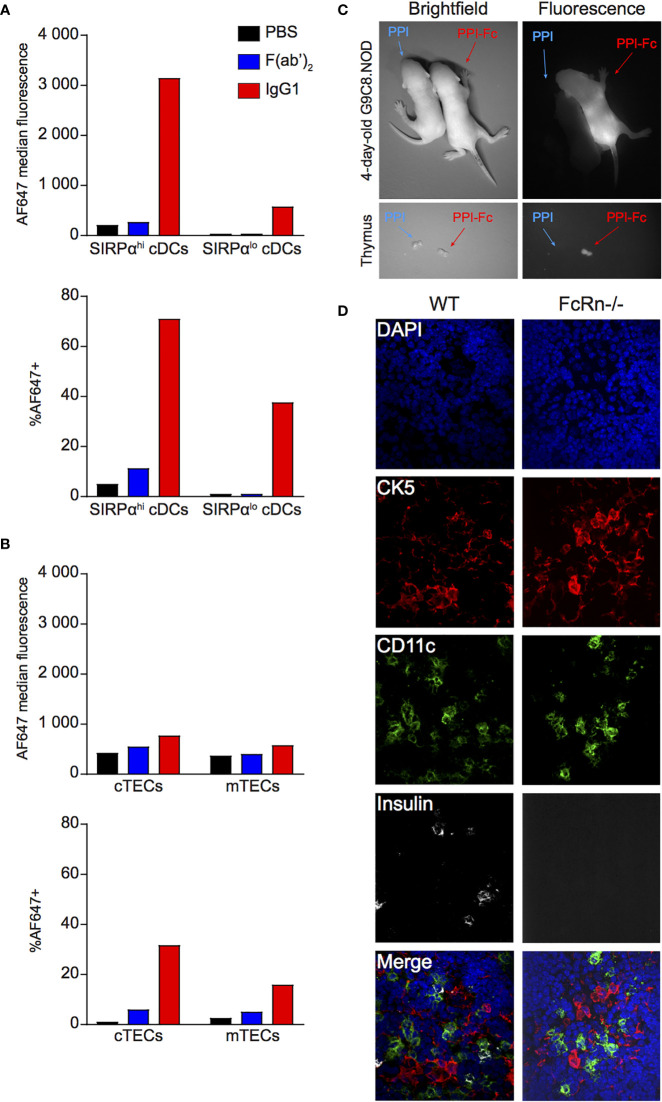
Oral PPI-Fc reaches the thymus mainly through ferrying by migratory SIRPα^hi^ cDCs. **(A, B)** Five-day-old G9C8.NOD mice were force-fed with 50 µg AF647-labeled IgG1 (red), F(ab’)_2_ (blue), or PBS (black). Thymi from 3 mice/group were pooled and cells were magnetically enriched for CD11c^+^ DCs **(A)** and EpCAM^+^ TECs **(B)** before flow cytometry (see **Supplementary Figure 5** for details). Protein uptake (AF647 median fluorescence and percentage of AF647^+^ cells) was analyzed in the indicated DC and TEC subsets. **(C)** *Ex-vivo* imaging of the whole body (top) and thymus (bottom) of 1-day-old G9C8.NOD mice 72 h after oral administration of 50 µg AF680-labeled PPI-Fc or PPI (day 4 of life). **(D)** Confocal images of thymic sections recovered from WT G9C8.NOD and FcRn^-/-^.B6 mice 24 h after oral administration of fluorescent PPI-Fc. Sections were stained for insulin (white), CD11c (green) and cytokeratin 5 (CK5, red), with nuclei counterstained with DAPI (blue).

We next investigated whether orally administered PPI-Fc can reach the thymus, as shown for the transplacental route (39). In the thymus (**Supplementary Figure 7A**), CD11c^hi^ cDCs can be divided into a SIRPα^lo^ and a SIRPα^hi^ subset, corresponding to resident and migratory thymic cDCs, respectively (39, 42, 43). Fluorescent signal was preferentially detected in migratory SIRPα^hi^ cDCs and was limited to IgG1 (70% AF647-labeled IgG1^+^ cells *vs* 10% ${\text{F}(\text{ab}’)}_{2}^{+}$; **Figure 5A**), suggesting that ferrying by cDCs is the predominant, although not exclusive, mechanism of Fc-protein delivery to the thymus.

We further analyzed the uptake by medullary (m)TECs (CD45^‒^EpCAM^+^UEA-1^+^) and cortical (c)TECs (CD45^‒^EpCAM^+^Ly51^+^) (**Supplementary Figure 7B**). Although detectable, their uptake was much more modest, and again limited to IgG1 (**Figure 5B**). As mTECs and cTECs are non-migratory, their IgG1 uptake suggests that Fc-coupled proteins gain access to the thymus also in soluble form, in line with the low-level signal detected in resident SIRPα^lo^ cDCs.

We verified by imaging whether the ferrying observed for IgG1 occurs also with PPI-Fc. Newborn G9C8.NOD mice analyzed 72 h after gavage displayed fluorescent PPI-Fc but not PPI signal both at the whole-body level and in isolated thymi (**Figure 5C**). Confocal microscopy on thymic tissue sections (**Figure 5D**) confirmed the presence of PPI-Fc fluorescence (white) associated with CD11c^+^ DCs (green) in WT G9C8.NOD mice, but not in their FcRn^-/-^.B6 counterparts. These CD11c^+^ DCs were competent for *in vitro* antigen processing and able to present PPI_B15-23_ peptide to G9C8 CD8^+^ T cells, inducing their activation and proliferation (**Supplementary Figure 8**).

Collectively, these results show that orally administered Fc-coupled proteins, but not Fc-devoid proteins, reach the thymus mostly through ferrying by migratory SIRPα^hi^ cDCs and, to a lesser extent, in soluble form.

### Oral PPI-Fc Partially Delays Diabetes Onset in Polyclonal *Ins2* ^-/-^.NOD Mice

**Figure 6:**
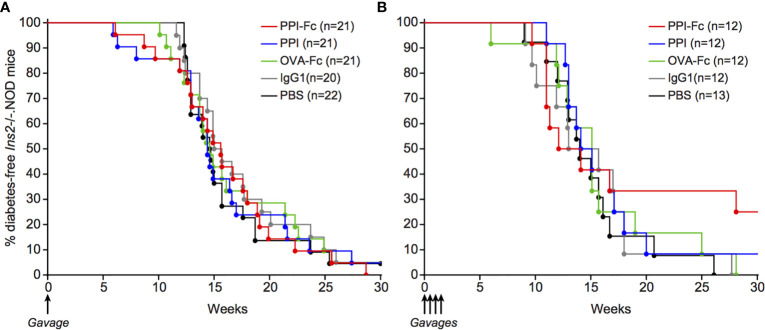
Oral PPI-Fc partially delays diabetes onset in polyclonal *Ins2* ^-/-^.NOD mice. Diabetes incidence in *Ins2* ^-/-^.NOD mice after oral administration of 50 µg PPI-Fc (red) or equimolar amounts of PPI (blue), OVA-Fc (green), IgG1 (grey) or PBS (black) at day 1 **(A)** or day 1, 4, 7 and 10 of life (**B**; p=0.13 between PPI-Fc and control groups by log-rank test).

Having defined that oral PPI-Fc crosses the gut epithelium, achieves systemic bioavailability in both soluble and APC-associated form and reaches the thymus, we reasoned that the lack of diabetes protection in G9C8.NOD mice may reflect the aggressive nature of this TCR-transgenic model. We therefore repeated these experiments in *Ins2* ^-/-^.NOD mice. This model is informative because it develops spontaneous diabetes and harbors a natural polyclonal T-cell repertoire. It thus provides a more physiological setting, in which oral PPI-Fc treatment should impact only the PPI-reactive T-cell fraction present in the natural repertoire. Moreover, *Ins2* knock-out leads to absent PPI expression in the thymus and lack of thymic deletion of PPI-reactive T cells, thus accelerating diabetes development (25, 26). The thymic delivery of orally administered PPI-Fc may thus compensate this defect and impact the overall autoimmune cascade downstream of PPI. We applied the same single-dose 50-µg regimen as before in 1-day-old *Ins2* ^-/-^.NOD mice. Also in this case, no diabetes protection was observed (**Figure 6A**). To verify whether this was due to suboptimal dosing, we attempted a more intensive regimen. Based on the kinetics of intestinal FcRn expression (**Figure 2A**), repeated 50-µg doses were administered at day 1, 4, 7 and 10 of life, in order to cover the time window displaying the highest intestinal FcRn expression. Again, no significant protection was obtained, but diabetes development was delayed by approximately 10 weeks in 33% of PPI-Fc-treated mice compared to control groups (**Figure 6B**).

Collectively, these data show that oral PPI-Fc, administered either in a single-dose or multiple-dose regimen, does not significantly prevent diabetes development in polyclonal *Ins2* ^-/-^.NOD mice, albeit a partial delay is observed with the multiple-dose regimen.

## Discussion

Most T1D preventative vaccination trials employed insulin in its native form, without modifications. When administered through the oral route (14, 44), this formulation holds several limitations. First, insulin is rapidly degraded in the acidic gastric environment under the action of several digestive enzymes. The little fraction that reaches the intestinal tract does not efficiently cross the epithelial barrier, thus resulting in minimal absorption, estimated at ~1-2% (14). Second, the use of insulin rather than its PPI precursor excludes several epitopes derived from the C peptide and, more importantly, the signal sequence (1). Our formulation employs an IgG1 Fc fragment, which is fused to PPI rather than insulin, thus providing a more comprehensive epitope coverage. Of further note, the affinity of proinsulin and PPI for the insulin receptor is much lower than that of insulin itself (5), thus minimizing undesirable metabolic side effects upon systemic absorption, e.g. hypoglycemia, which we did not observe. Although some gastric degradation likely takes place also for PPI-Fc, antibodies are less sensitive to such degradation (45), and the Fc moiety may increase PPI-Fc stability. Indeed, administration of a single 50-µg dose was sufficient to achieve intestinal and systemic bioavailability. Moreover, the Fc moiety can hijack the FcRn, which is highly expressed by intestinal epithelial cells, thus allowing PPI-Fc to cross the intestinal barrier. It is also likely that the Fc moiety may subsequently increase systemic half-life through the buffering effect of endothelial cells, which also uptake Fc-fused proteins *via* the FcRn (thus protecting them from degradation) and slowly release them back into the circulation (thus prolonging their bioavailability) (46). This buffering mechanism provides a rationale for several Fc-fused therapeutics (47).

Our observations show that the PPI-Fc formulation strategy is efficient in terms of antigen delivery in newborn mice, since the intestinal transfer is Fc- and FcRn-dependent, while such transfer and subsequent uptake by APCs is much less efficient for Fc-devoid PPI. Moreover, the expression of FcRs other than FcRn may facilitate PPI-Fc uptake by APCs, as we did not detect significant levels of FcRn on these cells. Indeed, PPI-Fc was mainly taken up by LP macrophages, which are known to express FcRs at high levels (48). Moreover, they might be more efficient than DCs to phagocytose PPI-Fc-loaded intestinal epithelial cells as part of their physiological apoptotic turnover (49–51). In macrophages, this uptake was high in both the CX3CR1^+^ and CX3CR1^‒^ subset. In CX3CR1^‒^ macrophages, uptake was superior in the SIRPα^hi^ than in the SIRPα^lo^ fraction. The functional differences between these two as yet undescribed subsets are unknown. PPI-Fc was also detected in LP cDCs, namely the CD103^+^SIRPα^‒^ and CD103^‒^SIRPα^+^ subsets. This uptake could be mediated by the FcRs that they also express, while other studies have shown that CD103^+^ cDCs are able to extend their dendrites through the epithelial gut layer to directly sample luminal antigens (52, 53). Transfer of antigens from macrophages to neighboring DCs through gap junctions may also occur (54). Albeit their uptake of Fc-coupled proteins was lower, these cDCs have migratory properties that are absent in macrophages, and may thus provide a ferrying system for PPI-Fc to reach different organs. Indeed, 70% of thymic SIRPα^hi^ cDCs were also loaded with Fc-coupled proteins. This cDC subset is described as migratory, i.e. reaching the thymus from the periphery, in contrast to SIRPα^lo^ cDCs that are described as thymic-resident (42, 43). It is thus likely that Fc-coupled proteins are ferried to the thymus by these migratory cDCs loaded in the periphery.

Although important, PPI-Fc ferrying by SIRPα^hi^ cDCs is not the only pathway allowing PPI-Fc to move across different body compartments, and several lines of evidence suggest that diffusion in soluble form is another mechanism at play. Indeed, spleen macrophages displayed the same uptake profile as in the LP, namely a higher uptake in the CX3CR1^+^ and the CX3CR1^‒^SIRPα^hi^ subsets compared to CX3CR1^‒^SIRPα^lo^ macrophages, which were virtually negative. Since the current consensus is that macrophages, including those of the LP, are non-migratory (55), this observation suggests that, following entry into the LP, PPI-Fc also circulates in soluble form and reaches the spleen, where it is taken up by resident macrophages. It should however be noted that mice were treated at 5 days of life, at a stage when the immune system is still under development and different tissues are being colonized by immune cells. The APC populations described herein could thus correspond to precursors or APCs at early differentiation stages that might still be endowed with migratory properties. In particular, CX3CR1 expression can mark the engagement of myeloid progenitors toward the monocyte/macrophage/DC lineage and is expressed by a common splenic macrophage/DC precursor (56). A splenic monocyte population expressing CD11b and CX3CR1 has also been described (56), possibly corresponding to the CX3CR1^+^ subset described herein, which was also CD11b^+^. Nonetheless, PPI-Fc was detected in the serum, thus providing direct evidence for its circulation in soluble form. In line with this interpretation, a sizable fraction of TECs (both mTECs and cTECs) also took up Fc-coupled proteins, likely mediated by the FcRn that they express (57). Although the transfer of peptide-MHC complexes from mTECs to DCs is described (58), no transfer in the opposite direction has been reported, arguing for a local uptake in soluble form. Finally, labeling of thymic DCs and mTECs by free AF647 dye is likely to be marginal, as both microscopy and flow cytometry experiments showed a clear fluorescent signal after oral administration of AF647-labeled PPI-Fc or IgG1, but not of control AF647-labeled PPI or F(ab’)_2_.

It is during the perinatal period that diabetogenic T cells escape negative selection (59), thus providing a critical time window for diabetes prevention. Moreover, the PPI-Fc uptake observed in the LP, spleen and thymus suggests that both peripheral and central tolerance mechanisms could be impacted with an optimized administration scheme. We previously showed that a single intravenous administration of PPI-Fc to pregnant mice protects their progeny from diabetes (39). Despite a promising profile in terms of systemic antigen availability and thymic delivery, the oral administration of this same protein to newborn mice did not achieve the same protection, nor did it modulate T-cell responses at early and late time points. The lack of therapeutic benefit is open to several explanations.

First, it may reflect an issue of timing, dosing or treatment duration. In our materno-fetal transfer model, the progeny was exposed to PPI-Fc even before birth, and PPI-Fc remained detectable for longer time, i.e. up to 7 days of life upon administration 2 days before birth (39). Serum PPI-Fc levels were lower (0.75 µg/ml *vs.* 2.2 µg/ml after oral administration), but remained stable during the first 48 h, while they rapidly decreased already at 24 h upon oral administration. The therapeutic and T-cell modulatory effects observed with the transplacental route and missed here could thus rely on the longer exposure to Fc proteins. Higher doses or repeated administrations may thus be needed to achieve diabetes prevention through the oral route. Indeed, the early peak and rapid decline of serum PPI-Fc concentration observed after oral administration may be less suited to induce tolerance compared with the lower but steadier serum concentration achieved after transplacental transfer. Alternatively, enteric-coated formulations may reduce degradation during the gastrointestinal transit. Treatment by *Lactococcus lactis* strains transgenically modified to release PPI-Fc, alone or in combination with modulatory cytokines such as IL-10 (60), is another attractive option, as it would obviate the need for PPI-Fc production, minimize gastrointestinal degradation, and achieve a more tolerogenic effect through prolonged delivery of small antigen amounts (60). Importantly the intestinal expression of FcRn is limited to the first 15 days of life in the mouse, but persists indefinitely in the human (61), thus widening the time window available for intervention. The availability of mouse strains with a human FcRn transgene under the control of its natural promoters (62) will be useful to explore prolonged treatment regimens, and to more closely mimic the interaction with the human FcRn ultimately targeted for clinical trials.

Second, the limitations of the G9C8.NOD model should be considered. Diabetes is induced by peptide immunization, thus resulting in a very aggressive autoimmune disease. Despite spontaneous diabetes onset, also the *Ins2* ^-/-^.NOD model is rather aggressive compared with WT NOD mice, as diabetes development is greatly accelerated (between 6 and 18 weeks of age). Although non-significant, a partial delay was observed after repeated oral PPI-Fc administration in *Ins2* ^-/-^.NOD newborn mice, suggesting that an optimized treatment regimen/formulation may be effective in WT NOD mice.

Third, the use of multiple antigens rather than PPI alone may be required to achieve a more comprehensive tolerogenic effect. This may be obtained by developing polypeptide Fc-fused constructs incorporating multiple epitopes targeted by autoimmune T cells. Oral combination treatments with other agents such as vitamin D3 (calcitriol) (63) or low-dose IL-2, e.g. IL-2-Fc muteins (64) that could be orally administered to exploit the same FcRn pathway, may also be considered.

In conclusion, the efficacy of oral PPI-Fc treatment in terms of systemic and thymic antigen delivery *via* the intestinal FcRn pathway warrants further investigations to develop antigen formulations and/or regimens that can translate into diabetes prevention.

## Prior Presentation

Parts of this study were presented at the congresses of the Immunology of Diabetes Society (IDS, London, October 25-29, 2018), and the Federation of Clinical Immunology Society (FOCIS, Boston, June 18-21, 2019).

## Data Availability Statement

The raw data supporting the conclusions of this article will be made available by the authors, without undue reservation.

## Ethics Statement

The animal study was reviewed and approved by The Ethics committee of Paris Descartes University and the French Ministry of Research.

## Author Contributions

Conceptualization, SY and RM. Methodology, NC, SC, SY, and RM. Investigation, NC, SC, CD, CL, SY, and RM. Data Curation, NC, SC, CD, SY, and RM. Writing, NC, CD, SY, and RM. Visualization, NC, SY, and RM. Supervision, SY and RM. Funding Acquisition, RM. RM is the guarantor of this work and, as such, had full access to all the data in the study and takes responsibility for the integrity of the data and the accuracy of the data analysis. All authors contributed to the article and approved the submitted version.

## Funding

This work was performed within the *Département Hospitalo-Universitaire* (DHU) AutHorS and supported by a PhD fellowship of the Ile-de-France CORDDIM and by grants from the JDRF (2-SRA-2016-203-S-B), the *Fondation Francophone pour la Recherche sur le Diabète*, the EFSD/JDRF/Lilly European Programme in Type 1 Diabetes Research 2015, the *Agence Nationale de la Recherche* (ANR-15-CE17-0018-01), Inserm-Transfert Proof of Concept 2016, the *Fondation pour la Recherche Médicale* (EQU20193007831), the *Association pour la Recherche sur le Diabète*; and the Innovative Medicines Initiative 2 Joint Undertaking under grant agreements 115797 and 945268 (INNODIA and INNODIA HARVEST), which receive support from the EU Horizon 2020 program, the European Federation of Pharmaceutical Industries and Associations, JDRF, and the Leona M. and Harry B. Helmsley Charitable Trust.

## Conflict of Interest

The authors declare that the research was conducted in the absence of any commercial or financial relationships that could be construed as a potential conflict of interest.

## Publisher’s Note

All claims expressed in this article are solely those of the authors and do not necessarily represent those of their affiliated organizations, or those of the publisher, the editors and the reviewers. Any product that may be evaluated in this article, or claim that may be made by its manufacturer, is not guaranteed or endorsed by the publisher.
