# Supplementary material for: Oral Fc-Coupled Preproinsulin Achieves Systemic and Thymic Delivery Through the Neonatal Fc Receptor and Partially Delays Autoimmune Diabetes
Source: Front Immunol. 2021 Aug 10;12:616215. doi: 10.3389/fimmu.2021.616215 (PMC8382691; doi:10.3389/fimmu.2021.616215)
Supplement: Supplementary file 1 [file DataSheet_1.pdf]

Supplementary Material

1 Supplementary Methods

1.1 Isolation of intestinal epithelial cells

The digestive tract (from duodenum to rectum, excluding cecum) was dissected from G9C8.NOD mice of different ages and from 1-day-old FcRn$^{-/-}$.B6 mice. After removal of adipose tissue and Peyer's patches, tissues were washed with cold PBS, minced and transferred into complete RPMI, supplemented with 10% FBS, 2 mM L-Glutamine, 1 mM sodium pyruvate, 1X non-essential amino-acids and antibiotics (100 U/ml penicillin, 100 µg/ml streptomycin, 25 µg/ml enrofloxacin, 100 U/ml polymyxin B; PSEP mix). Tissue was cut into 1.5 cm pieces and agitated at 200 rpm at 37°C for the following incubation steps:

- 20 ml HBSS-DTT (HBSS, 5mM DTT, 2% FBS, PSEP) for 20 min;
- 30 ml HBSS-EDTA (HBSS, 5 mM EDTA, 2% FBS, PSEP), thrice for 15 min.

The supernatants containing intestinal epithelial cells were pooled and centrifuged at 600g for 5 min.

1.2 Tissue preparation for cell isolation and flow cytometry

For intestinal LP preparations, the digestive tract was processed as above. Following the last HBSS-EDTA washing step, the remaining tissue was incubated in 10 ml HBSS-HEPES (HBSS, 10 mM HEPES, PSEP) for 10 min. Pieces were then transferred into a Petri dish filled with 10 ml HBSS-HEPES, minced, transferred into a 15-ml tube containing 7 ml of a digestion solution (HBSS, 10 mM HEPES, PSEP, 0.2 Wünsch Units/ml Liberase, 200 Kunitz Units/ml DNase) and incubated at 37°C for 30 min under agitation. The reaction was stopped by adding 5 ml of complete RPMI and tissue pieces were passed through a 70-µm strainer. After centrifugation at 600g for 5 min and two washes in 15 ml HBSS supplemented with 2% FBS, cell pellets were resuspended in PBS and stained.

Spleen, mesenteric (MLN) and pancreatic lymph node (PLN) cells were filtered through a 40-µm strainer, centrifuged at 600g for 10 min. Splenocytes were resuspended in 1 ml hemolytic buffer for 3 min on ice. After washing in complete RPMI, cells were filtered again and kept on ice for subsequent analysis.

Thymi were cut into small pieces in RPMI supplemented with 10 mM HEPES and incubated thrice at room temperature for 15 min under agitation (200 rpm). Thymocyte-containing supernatants were discarded. The pellet was laid onto a 70-µm cell strainer placed in a well of a 6-well plate, and incubated with 2 ml of RPMI/HEPES containing collagenase IV (22 U/ml; Serlabo) for 15 min at 37°C under agitation. The supernatant was collected and the remaining tissue was further incubated thrice for 15 min at 37°C under agitation in 2 ml of RPMI/HEPES containing 22 U/ml collagenase IV, 0.6 U/ml neutral protease (Serlabo) and 50 Kunitz/mL DNAse I (Sigma). Supernatants from these digestion steps were pooled and incubated with anti-CD11c magnetic beads (Miltenyi Biotec) for DC isolation. The CD11c^{-} fraction was then incubated with anti-EpCAM magnetic beads (Miltenyi #130-105-958) to isolate thymic epithelial cells.

1.3 Antigen presentation assays on neonatal thymic DCs

DCs were isolated from the thymi of 5-10-day-old G9C8.NOD mice (n=10) using CD11c microbeads (Miltenyi), and incubated with 0.5 µg of $PPI_{B15\text{-}23}$ or $PPI_{B9\text{-}23}$ peptide or vehicle overnight (16-18 h) before addition at a 1:1 ratio of CFSE-labeled T cells isolated from the spleen of adult G9C8.NOD mice (MojoSort CD3 T cell isolation kit, BioLegend). Cells were co-cultured for 3 days in the presence of 20 U/ml recombinant murine IL-2 (R&D). CFSE dilution and CD25/CD69 expression was assessed by flow cytometry.

2 Supplementary Tables

Marker	Fluorochrome	Clone	Manufacturer	Dilution
**Live/DEAD**	Aqua	/	Invitrogen	1/1,000
**NK1.1**	BV510	PK136	BD Biosciences	1/100
**CD3**	BV510	145-2C11	BD Biosciences	1/100
**CD45**	BV786	30-F11	BD Biosciences	1/200
**F4/80**	BV711	BM8	BioLegend	1/50
**B220**	PE-Cy7	RA3-6B2	BD Biosciences	1/150
**SiglecF**	AF488	1RNM44N	eBioscience	1/50
**CD11c**	BV605	N418	BioLegend	1/50
**CD11b**	BV650	M1/70	BD Biosciences	1/150
**CX3CR1**	PE	5A011F11	BioLegend	1/200
**CD103**	PE-CF594	M290	BD Biosciences	1/200
**SIRPα**	PerCP-eFluor710	P84	eBioscience	1/50
**PDCA-1**	Pacific Blue	927	BioLegend	1/200
**FcRn**	Unconjugated	/	R&D Systems	1/50
**Anti-goat IgG**	PE	/	R&D Systems	1/100

Supplementary Table 1. APC antibody panel.

Marker	Fluorochrome	Clone	Manufacturer	Dilution
**Live/DEAD**	Aqua	/	Invitrogen	1/1,000
**NK1.1**	BV510	PK136	BD Biosciences	1/100
**CD3**	BV510	145-2C11	BD Biosciences	1/100
**CD45**	BV786	30-F11	BD Biosciences	1/200
**EpCAM**	APC-Cy7	G8.8	BioLegend	1/50
**UEA-1**	FITC	/	Vector Labs	1/25
**Ly51**	PE	6C3	BioLegend	1/100

Supplementary Table 2. TEC antibody panel.

Marker	Fluorochrome	Clone	Manufacturer	Dilution
**Live/DEAD**	Aqua	/	Invitrogen	1/1,000
**TCR Vβ6**	PerCP-eFluor710	145-2C11	eBioscience	1/100
**CD4**	BV711	RM4-5	BD Biosciences	1/200
**CD8**	AF700	53-6.7	BD Biosciences	1/150
**CCR9**	PE-Cy7	CW-1.2	BioLegend	1/200
**LPAM-1**	PE-CF594	DATK32	BD Biosciences	1/100
**CD44**	BV786	IM7	BD Biosciences	1/200
**CD62L**	BV605	MEL-14	BD Biosciences	1/200
**NRP1**	APC	3E12	BioLegend	1/50
**Foxp3**	PE	FJK-16s	eBioscience	1/50

Supplementary Table 3. T-cell antibody panel.

Marker	Fluorochrome	Clone	Manufacturer	Dilution
**CD107a**	APC	1D4B	BioLegend	1/400
**Live/DEAD**	Aqua	/	Invitrogen	1/1,000
**TCR Vβ6**	PerCP-eFluor710	145-2C11	eBioscience	1/100
**CD8**	BV711	53-6.7	BD Biosciences	1/150
**CD4**	BV421	GK1.5	BD Biosciences	1/200
**CD69**	PE-Cy7	CW-1.2	BioLegend	1/200
**IFN-γ**	PE	XMG1.2	BD Biosciences	1/50
**TNF-α**	FITC	MP6-XT22	BD Biosciences	1/50

Supplementary Table 4. Recall antibody panel.

3 Supplementary Figures

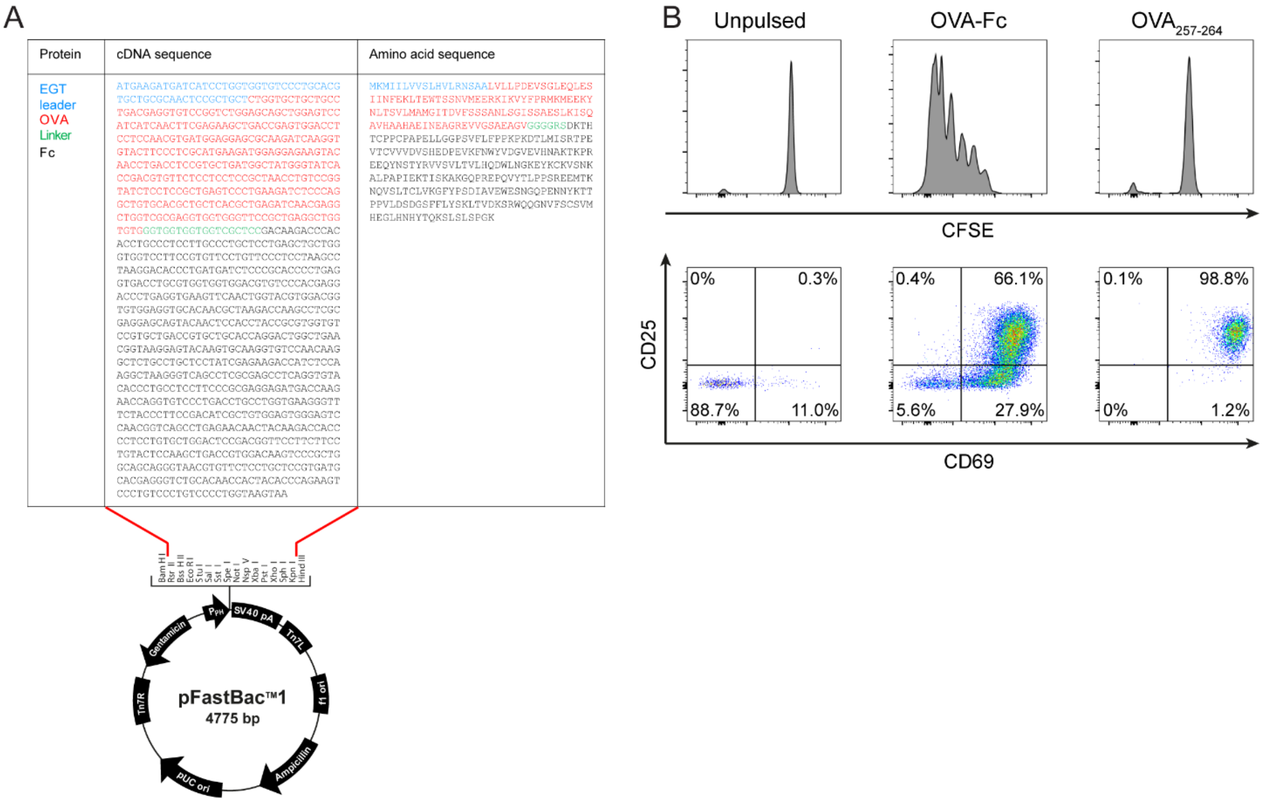

Supplementary Figure 1. OVA-Fc construct and validation of the Ova-Fc recombinant protein. (**A**) cDNA and amino-acid sequence of the OVA-Fc construct. An EGT insect cell secretion signal sequence (blue) was added at the 5'/N-terminal end. The construct was inserted into the pFastBac1 Baculovirus plasmid between BamHI and HindIII restriction sites. (**B**) *In vitro* validation of OVA-Fc fusion protein. Bone marrow–derived DCs prepared from naïve G9C8 mice were pulsed or not with 13 µM OVA-Fc or $OVA_{257-264}$ (SIINFEKL) peptide, and then matured with lipopolysaccharide prior to culture with CFSE-labeled OT-I cells for 5 days. CFSE profiles (top panels) and CD69/CD25 expression (bottom panels) are shown after gating on $CD8^+$ T cells.

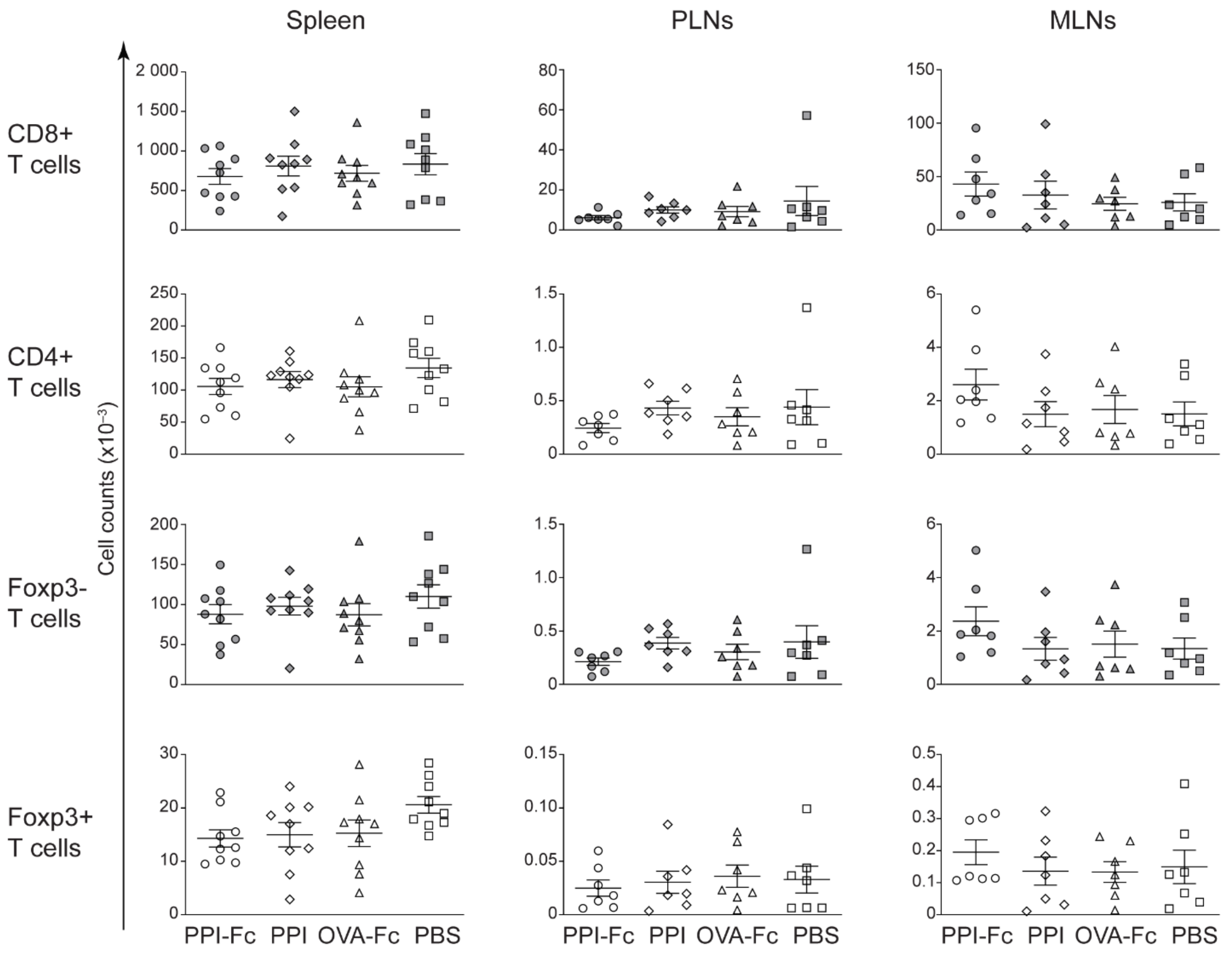

Supplementary Figure 2. Absolute numbers of $CD8^+$ and $CD4^+$ T cells in PPI-Fc-treated G9C8.NOD mice. Absolute counts ($x10^{-3}$) of $CD8^+$ and $CD4^+$ T cells (first and second row) and of $CD4^+Foxp3^-$ and $CD4^+Foxp3^+$ T cells (third and fourth row) in the spleen (left), PLNs (middle) and MLNs (right) of 4-week-old PPI-Fc- and control-treated G9C8.NOD mice. These mice are the same as those depicted in Figure 1B-C-D.

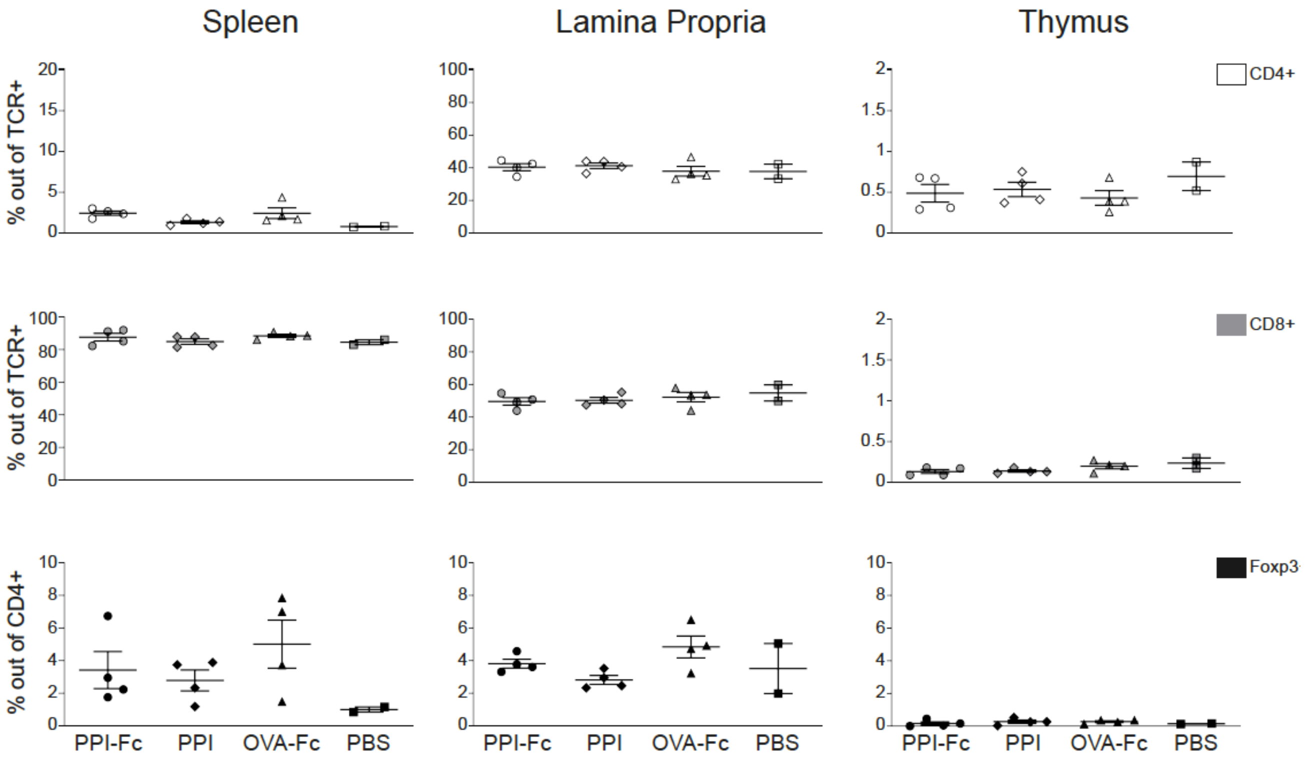

Supplementary Figure 3. Frequency of CD8^{+}, CD4^{+} and Foxp3^{+} T cells in PPI-Fc-treated G9C8.NOD newborns. Percent of CD8^{+} and CD4^{+} T cells (gated on TCR^{+} cells, first and second row) and of CD4^{+}Foxp3^{+} T cells (gated on CD4^{+} T cells, third row) in the spleen (left column), *lamina propria* (middle column) and thymus (right column) of 7-day-old G9C8.NOD mice treated at day 1 of life with oral PPI-Fc- or control proteins.

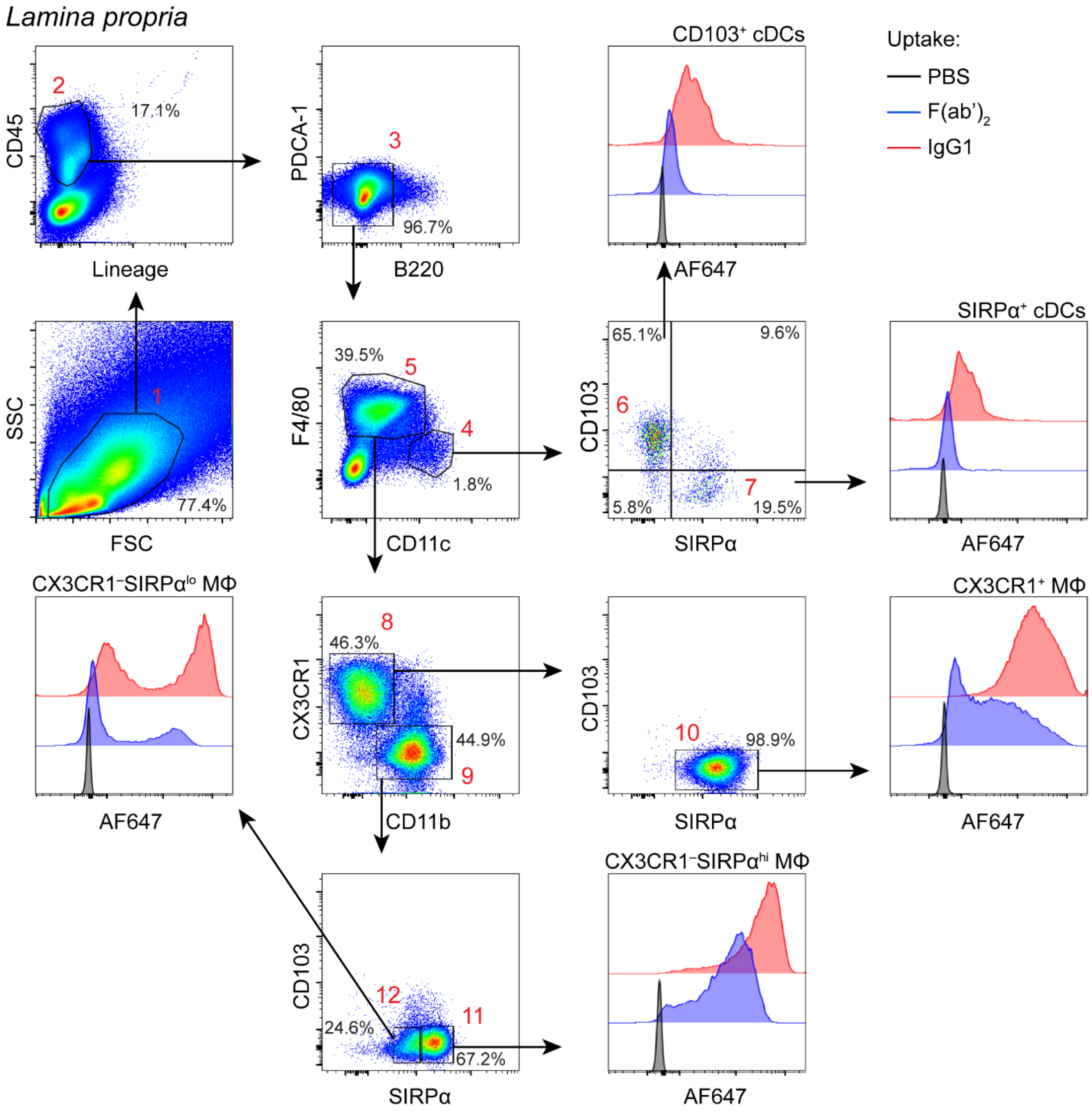

Supplementary Figure 4. Identification of intestinal LP APC subsets and fluorescent protein uptake by flow cytometry. Red numbers indicate sequential gates. After a first selection based on FSC/SSC morphology (1), $CD45^+Lineage^-$ ($CD3/NK1.1^-$) (2) and $B220^-PDCA\text{-}1^-$ cells (3) were gated. DCs were defined as $CD11c^{hi}$ (4), and subdivided into $CD103^+SIRP\alpha^-$ (6) and $SIRP\alpha^+CD103^-$ (7). Macrophages (MΦ) were selected by gating on $F4/80^+$ cells (5) and subdivided into $CX3CR1^+CD11b^-$ (8) and $CXCR1^-CD11b^+$ (9). $CX3CR1^+CD11b^-$ macrophages were all $SIRP\alpha^+$ (10), while the $CX3CR1^-CD11b^+$ subset was further subdivided into $SIRP\alpha^{hi}$ (11) and $SIRP\alpha^{lo}$ (12). The AF647-labeled protein uptake by each APC population is depicted in histograms.

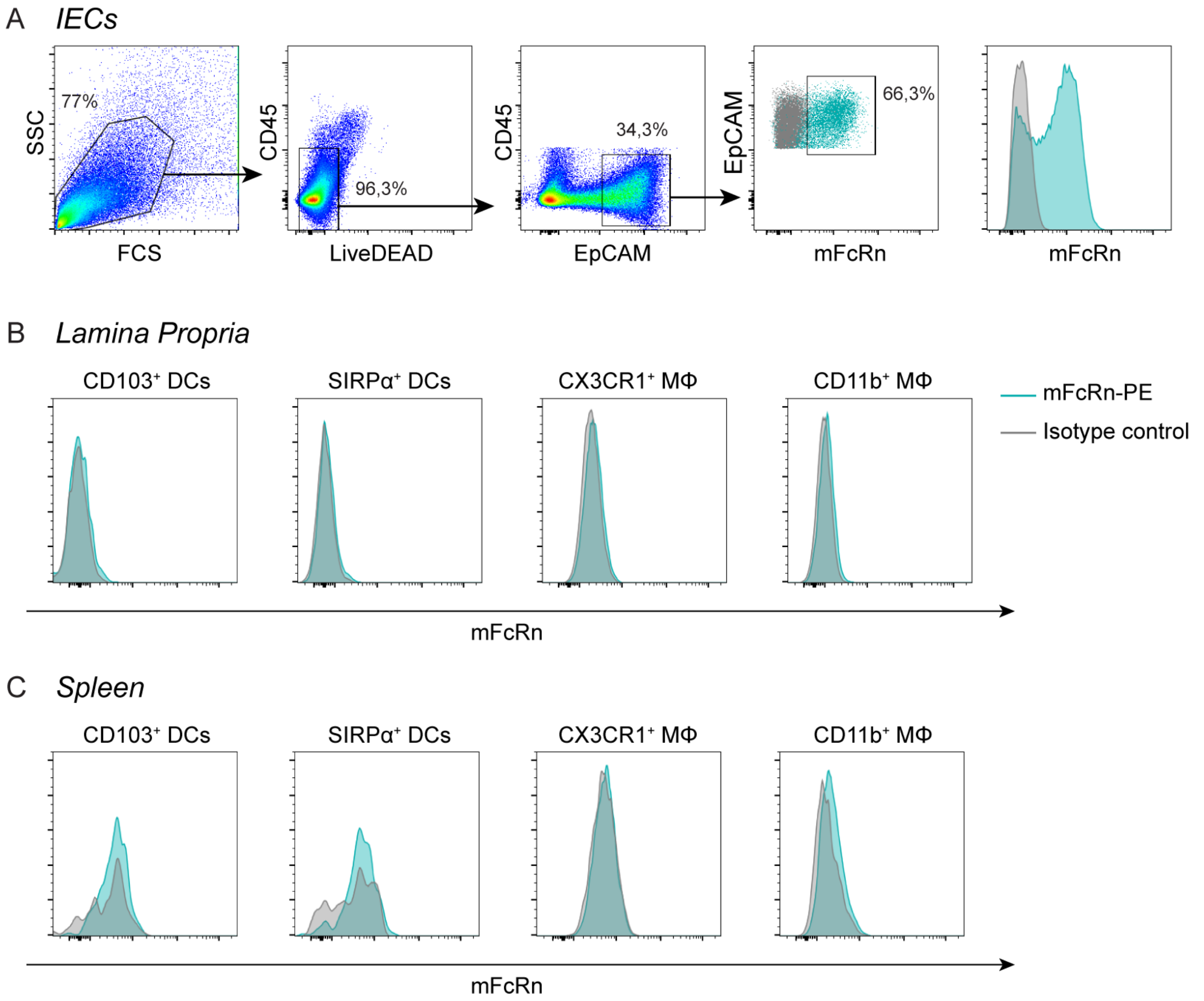

Supplementary Figure 5. FcRn is highly expressed in intestinal epithelial cells, but not in APCs in newborn mice. FcRn expression was analyzed in $CD45^{-}EpCam^{+}$ intestinal epithelial cells (IECs, **A**), as well as in $CD103^{+}$ and SIRPα^{+} DCs, and $CX3CR1^{+}$ and $CD11b^{+}$ macrophages (MΦ) from the *lamina propria* (**B**) and spleen (**C**) of newborn G9C8.NOD mice.

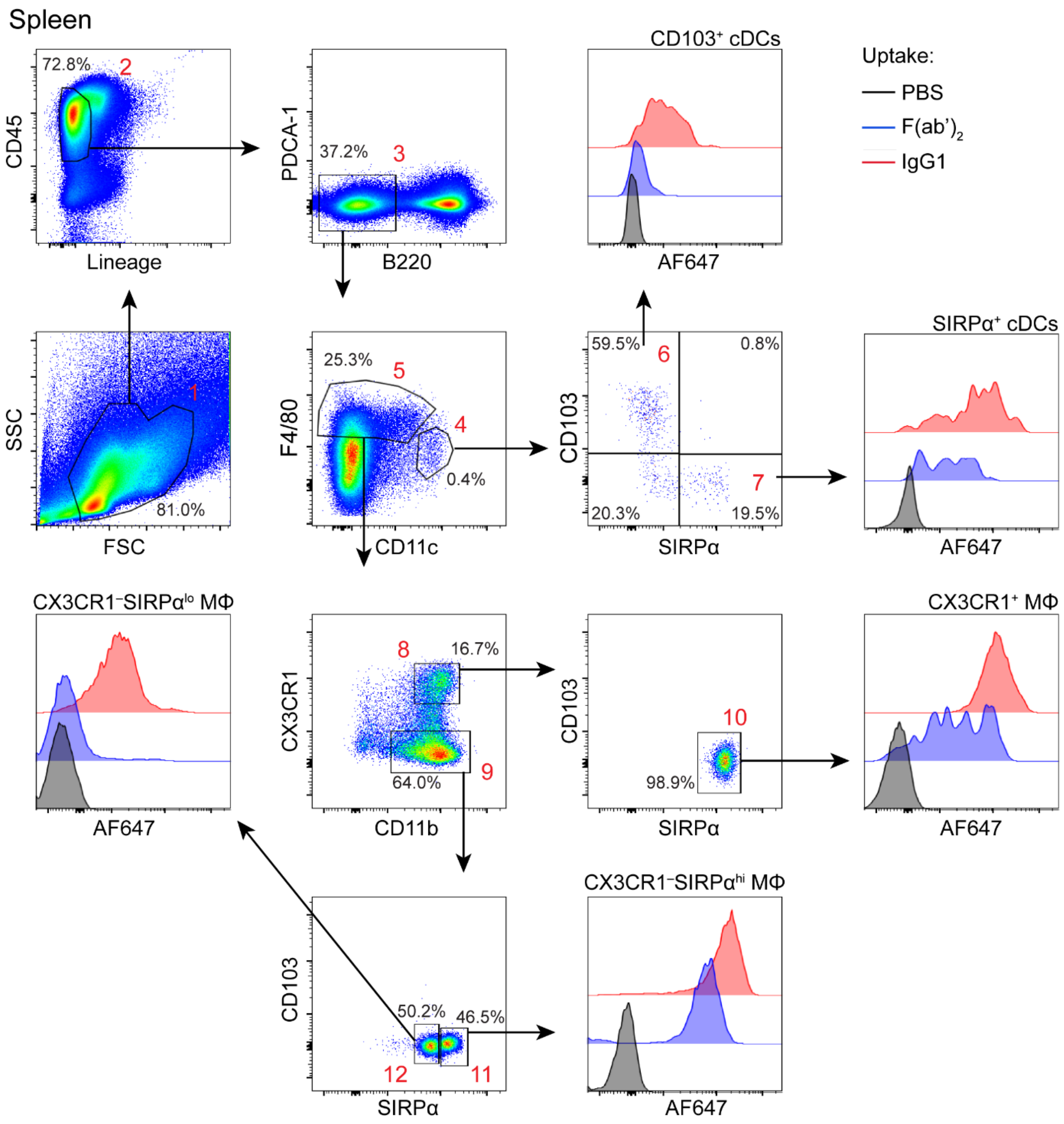

Supplementary Figure 6. Identification of splenic APC subsets and fluorescent protein uptake by flow cytometry. Red numbers indicate sequential gates. After a first selection based on FSC/SSC morphology (1), $CD45^{+}Lineage^{-}$ ($CD3/NK1.1^{-}$) (2) and $B220^{-}PDCA\text{-}1^{-}$ cells (3) were gated. DCs were defined as $CD11c^{hi}$ (4), and subdivided into $CD103^{+}SIRP\alpha^{-}$ (6) and $SIRP\alpha^{+}CD103^{-}$ (7). Macrophages (MΦ) were selected by gating on $F4/80^{+}$ cells (5) and subdivided into $CX3CR1^{+}CD11b^{+}$ (8) and $CXCR1^{-}CD11b^{+}$ (9). $CX3CR1^{+}CD11b^{+}$ macrophages were all $SIRP\alpha^{+}$ (10), while the $CXCR1^{-}CD11b^{+}$ subset was further subdivided into $SIRP\alpha^{hi}$ (11) and $SIRP\alpha^{lo}$ (12). The AF647-labeled protein uptake by each APC population is depicted in histograms.

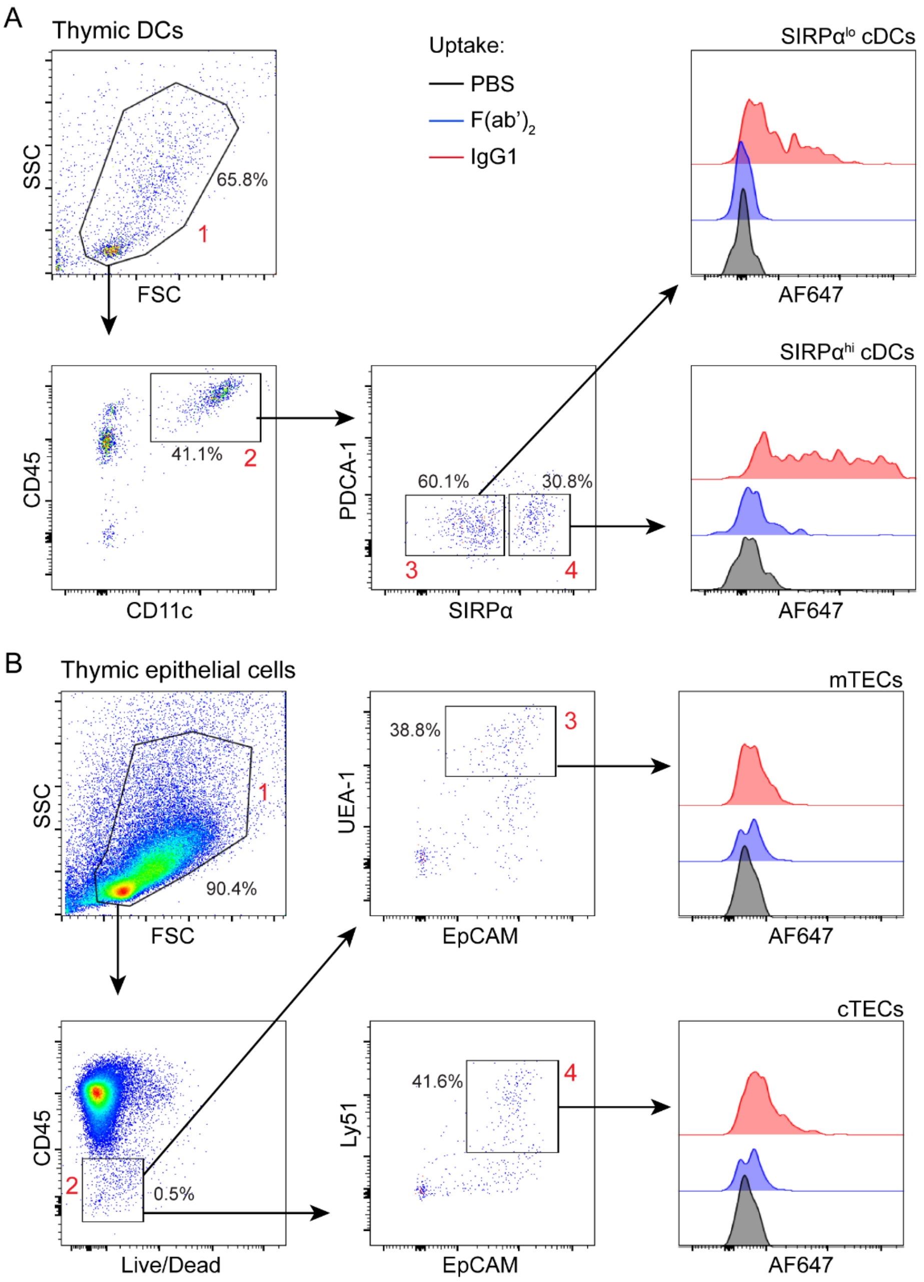

Supplementary Figure 7. Identification of thymic DC and epithelial cell subsets and fluorescent protein uptake by flow cytometry. Red numbers indicate sequential gates. (**A**) Thymic DCs. After a first selection based on FSC/SSC morphology (1), live $CD45^{+}CD11c^{hi}$ DCs (2; Live/Dead gate not shown) were gated and subdivided into $SIRP\alpha^{lo}$ (3) and $SIRP\alpha^{hi}$ (4). The AF647-labeled protein uptake by each DC population is depicted in histograms. (**B**) Thymic epithelial cells (TECs). After a first selection based on FSC/SSC morphology (1), live $CD45^{-}$ cells (2) were gated and subdivided into mTECs ($EpCAM^{+}UEA\text{-}1^{+}$, 3) and cTECs ($EpCAM^{+}Ly51^{+}$, 4). The AF647-labeled protein uptake by each TEC population is depicted in histograms.

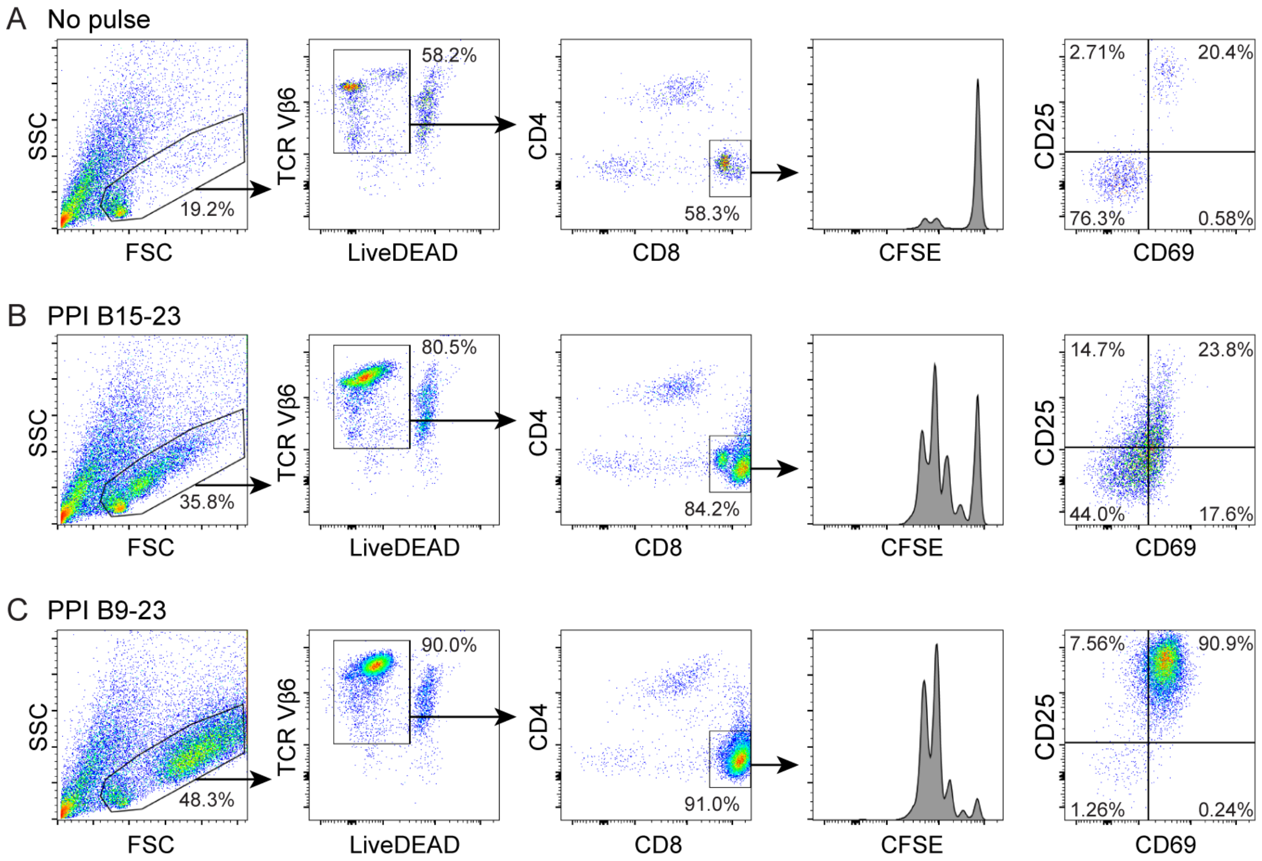

Supplementary Figure 8. Antigen processing and presentation of PPI peptides by neonatal thymic DCs. Proliferation and upregulation of the CD25 and CD69 activation markers were analyzed in CFSE-labeled splenic G9C8.NOD T cells after a 3-day culture with thymic DCs isolated from 5-10-day-old G9C8.NOD mice and pulsed overnight with peptide vehicle (**A**), $PPI_{B15\text{-}23}$ (**B**) or $PPI_{B9\text{-}23}$ peptide (**C**). After a first gating based on FSC/SSC morphology, viable TCR-Vβ6^{+} T cells were divided into CD4^{+} and CD8^{+} subsets. CFSE dilution and CD25/CD69 expression were analyzed in the CD8^{+} gate.
